# Shikonin Inhibits the Proliferation of Human Lens Epithelial Cells by Inducing Apoptosis through ROS and Caspase-Dependent Pathway

**DOI:** 10.3390/molecules19067785

**Published:** 2014-06-11

**Authors:** Wan-Rong Huang, Yue Zhang, Xin Tang

**Affiliations:** Tianjin Eye Hospital, Clinic College of Ophthalmology, Tianjin Medical University, Tianjin 300020, China; E-Mails: scarlethwang@aliyun.com (W.-R.H.); zmoon0225@126.com (Y.Z.)

**Keywords:** human lens epithelial cells, posterior capsular opacification, apoptosis, caspase-dependent pathway

## Abstract

Shikonin is a compound from the herbal plant *Lithospermum erythrorhizon* that has been proved to possess powerful anti-proliferative effect on many kinds of cancers and to be safe in *in vivo* study. Posterior capsular opacification (PCO), the most frequent complication of cataract surgery, is mainly caused by the uncontrolled proliferation of retained human lens epithelial cells (HLEs). In this study, we investigated the effect of shikonin on the proliferation of HLEs and explored its underlying mechanism of action. Shikonin significantly inhibited the proliferation of HLEs in a dose- and time-dependent manner. Its anti-proliferative effect was exerted through induction of apoptosis. Reactive oxygen species (ROS) generation played an essential role in this apoptotic process. Interestingly, scavenging of ROS completely blocked the apoptosis induced by shikonin. In addition, the treatment of shikonin in HLEs significantly increased the ratio of Bax/Bcl-2, disrupted mitochondria membrane potential (MMP) and activated caspases. The inhibition of caspase largely blocks the apoptosis. The changes of MAPK pathway were also demonstrated. Shikonin effectively inhibited the phosphorylation of ERK, while it activated the phosphorylation of JNK and p38. These results suggested that shikonin inhibited the proliferation of HLEs by inducing apoptosis through ROS generation and the caspase-dependent pathway and the MAPK pathway was also involved.

## 1. Introduction

PCO is the most common complication of cataract surgery [1]. The prevalence five years after surgery has been reported as 30–40% in adults and 100% in children [2]. PCO is caused by retained human lens epithelial cells in the capsular bag following surgery which then undergo proliferation, migration, epithelial-mesenchymal transition, collagen deposition, and lens fiber generation [3]. Up to now, the main ways to prevent PCO have centered on surgical techniques, laser treatment [4], pharmacological methods [5,6,7] and molecular biotechnology [8,9,10,11] to remove or destroy HLEs. However, the surgical way or Nd:YAG (neodymium-doped yttrium aluminum garnet; Nd:Y_3_A_l5_O_12_) laser treatment have a high risk of development of vision-related complications and are very expensive [12]. Pharmacological agents or small molecules have been used experimentally to prevent PCO, but their low efficacy and serious side effect remains an unsolved problem. Therefore, to find new effective and safe methods or agents with minimal side effects to prevent PCO is very meaningful and urgent.

Shikonin is a naphthoquinone derived from the root of *Lithospermum erythrorhizon*, and it has been widely used in China for centuries for its anti-inflammation properties and the treatment of external wounds [13]. The results from a clinical study using a shikonin-containing mixture demonstrated its safety and efficacy for the treatment of late-stage lung cancer patients [14]. It has been proved to possess powerful anti-proliferative effect on many kinds of cancers [15,16,17], but the efficacy on HLEs remains unknown.

In this study, we examined anti-proliferative effect of shikonin on HLEs and attempted to explore its underlying mechanism of action. The changes of cells in apoptosis, MMP and ROS production were examined by flow cytometry after staining with Annexin V/propidium iodide (PI), JC-1 (5,5',6,6'-tetrachloro-1,1',3,3'-tetraethylbenzimidazocarbocyanine iodide) and CM-H_2_DCFDA (chloromethyl-2',7'-dichlorodihydrofluorescein diacetate) respectively.

## 2. Results

### 2.1. Shikonin Inhibited the Proliferation of HLEs

**Figure 1:**
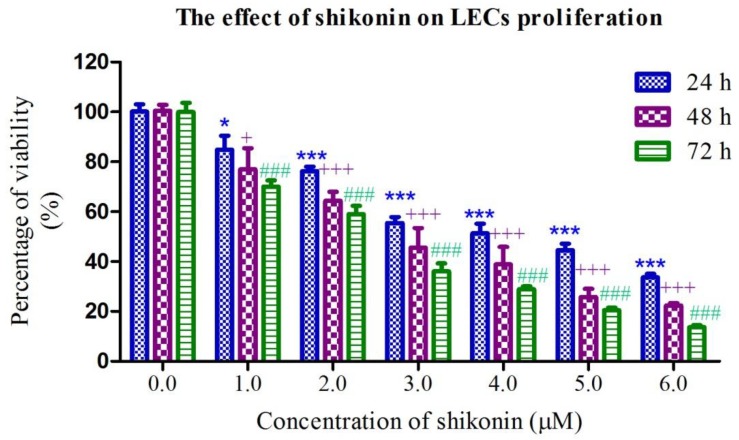
Shikonin significantly inhibited the cells proliferation of HLEs. Cells were treated with different concentration of shikonin for 24, 48 and 72 h. Results are expressed as mean ± S.E.M. (*n* = 3, * *p* < 0.05, ** *p* < 0.01, *** *p* < 0.001 for 24 h analysis; + *p* < 0.05, ++ *p* < 0.01, +++ *p* < 0.001 for 48 h analysis; # *p* < 0.05, ## *p* < 0.01, ### *p* < 0.001 for 72 h analysis).

To investigate the anti-proliferative effect of shikonin on HLEs, HLE B-3 cells were exposed to different concentrations of shikonin for 24, 48 and 72 h. Then the inhibition of proliferation was determined by an MTT assay. As shown in Figure 1, the inhibitory effect of shikonin on the growth of HLEs occurs in a dose-dependent manner. The IC_50_ value of shikonin on HLE B-3 at 24, 48 and 72 h is 4.58 ± 0.76 μM, 2.47 ± 0.52 μM and 1.86 ± 0.33 μM respectively. These suggested that shikonin significantly inhibited the proliferation of HLE B-3 cells.

### 2.2. Shikonin Induced Apoptosis in HLEs

**Figure 2:**
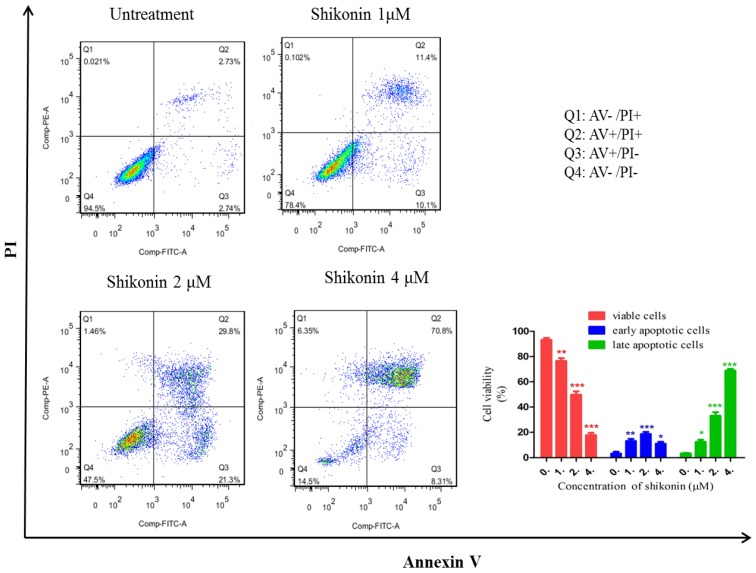
Shikonin induced apoptosis in HLEs at different concentration for 24 h. After treated with shikonin (0, 1, 2, 4 μM) for 24 h, cells were collected for apoptosis analysis. As treated with the higher concentration of shikonin, the percentage of apoptotic cells (Q2 + Q3) was gradually increased. * *p* < 0.05, ** *p* < 0.01, *** *p* < 0.001.

To investigate whether shikonin exerts its inhibitory effect by inducing apoptosis in HLEs, an Annexin V/PI double staining assay was applied. Annexin V was used to detect phosphatidylserine externalization, which is a hallmark of the early phase of apoptosis. PI on the other hand is a label of DNA fragments, a symbol of cell death. As shown in Figure 2, in the shikonin untreated group, 94.5% cells were gathered together at Q4 area (both Annexin V and PI are negative), which represents that cells are intact and healthy. Q2 and Q3 represent that cells are undergoing early and late apoptosis. The percentage of apoptotic cells (Q2 + Q3) in the non-treatment group was less than 6%. However, after the treatment of shikonin, even in 1 μM group, only 78.4% cells remained in Q4 area. Correspondingly, the percentage of cells in Q2 and Q3 was significantly increased, which represents the late and early apoptosis. In 2 μM group, there is less than 50% cells remained in Q4 area, and in 4 μM group, around 80% of cells went to Q2 and Q3 areas. Therefore, these results indicate that shikonin can significantly induce apoptosis in HLEs.

### 2.3. Shikonin Induced Apoptosis Through Mitochondrial and Caspases Dependent Pathway

**Figure 3:**
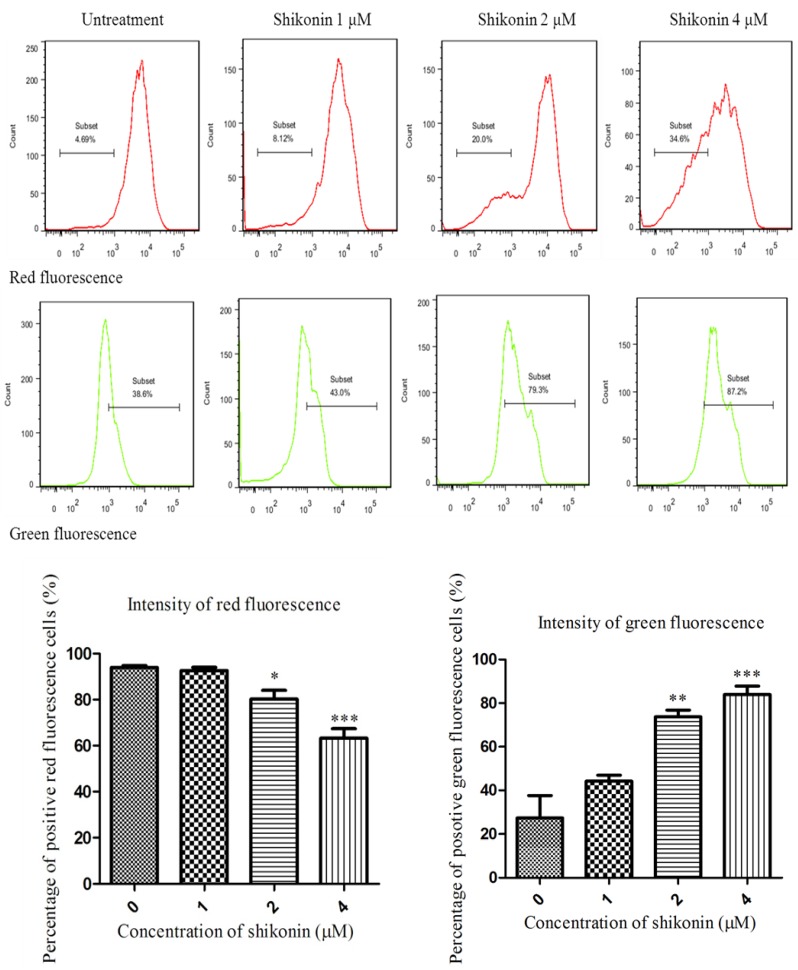
Shikonin induced the loss of MMP in HLEs. Flow cytometry analysis of the level of MMP of HLEs after the treatment of shikonin for 24 h. Compared with the untreated group, the shikonin treated group showed a significant loss of MMP, which was indicated by the higher green fluorescence but lower red fluorescence. * *p* < 0.05, ** *p* < 0.01, *** *p* < 0.001.

**Figure 4:**
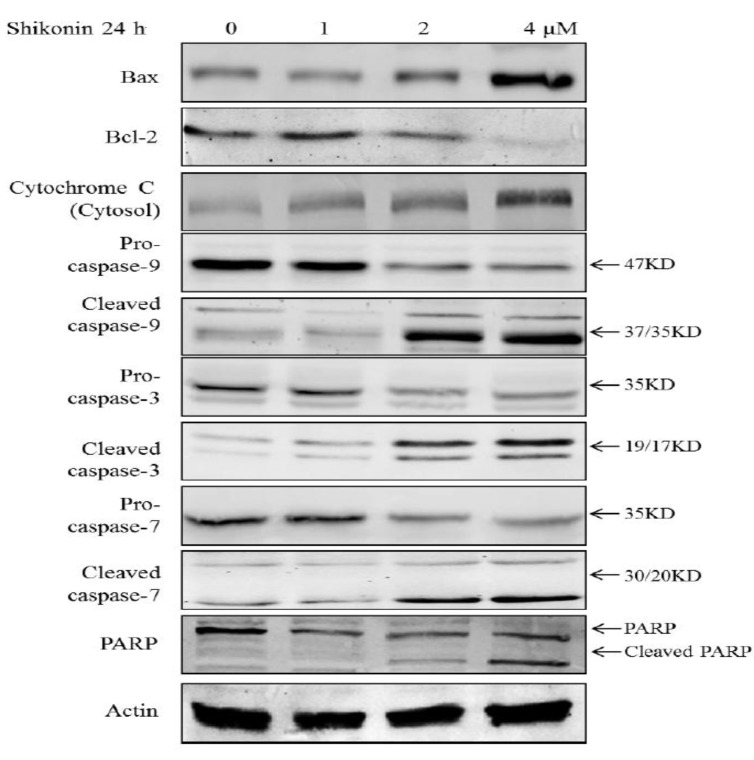
The anti-apoptotic protein Bcl-2 was significantly decreased while the pro-apoptotic protein Bax was largely increased by the treatment of shikonin. The key regulator of apoptosis caspase-3, -7 and 9 were activated and PARP was cleaved. Moreover, Cytochrome C was released from mitochondria to the cytosol.

To determine whether shikonin-induced apoptosis is through a mitochondria dependent pathway, the MMP of cells was detected with the mitochondria-sensitive dye, JC-1 [18]. As shown in Figure 3, compared with the untreated group, in the shikonin treated group, more JC-1 monomer was formed which emits green fluorescence, indicating that mitochondria were gradually losing MMP. On the other hand, less JC-1 accumulates in mitochondria where it emits a red fluorescence. The results suggest that shikonin induced the loss of MMP in HLE B-3 cells. The loss of MMP would inevitably result in the damage of the mitochondrial membrane. Therefore, we measured the proteins associated with the mitochondrial apoptotic pathway: Bcl-2 and Bax. The ratio of Bcl-2/Bax is considered as the indicator of the integrity of mitochondria membrane. As shown in Figure 4, after treatment with shikonin, the ratio of Bcl-2/Bax significantly decreased, which means that the mitochondrial membranes were disrupted. Therefore, both MMP analysis and western blot results provided evidence that mitochondria were involved in shikonin-induced apoptosis.

**Figure 5:**
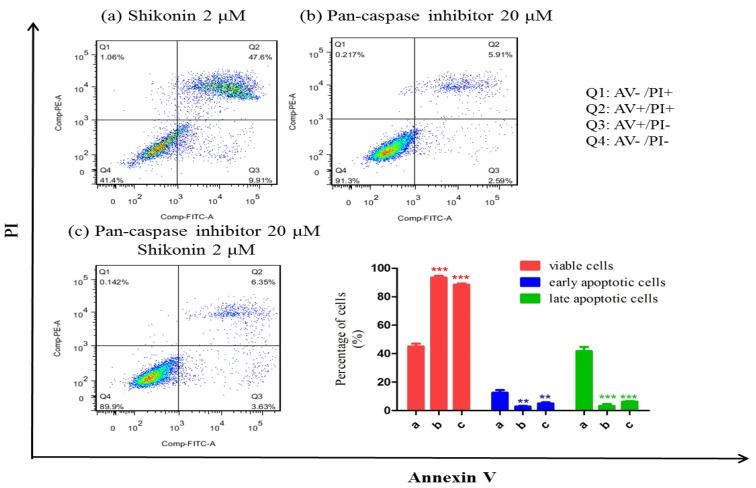
Pan-caspase inhibitor can mostly inhibit the apoptosis induced by shikonin. (**a**) single shikonin 2 μM treatment group. (**b**) Single pan-caspase inhibitor 20 μM treatment group. (**c**) shikonin 2 μM and pan-caspase inhibitor 20 μM co-treatment group. * *p* < 0.05, ** *p* < 0.01, *** *p* < 0.001.

Moreover, the activation of caspases was also detected. As shown in Figure 4, both caspase-3 and 7, 9 were activated by the treatment of shikonin in HLE B-3; its downstream target PARP was cleaved to become activated form; Cytochrome C was released from mitochondria. These results indicate that the apoptosis was mediated through mitochondrial intrinsic cell death pathway. To ascertain whether the activation of caspases is required for the apoptosis induced by shikonin, the pan-caspase inhibitor was applied to co-treat with shikonin for 24 h. As shown in Figure 5, compared with the shikonin single treatment group, the group co-treated with pan-caspase inhibitor and shikonin showed remarkably inhibited apoptosis induced by shikonin, which means that caspases activation is required for the apoptosis.

### 2.4. ROS Generation was Required for Shikonin-Induced Apoptosis in HLEs

**Figure 6:**
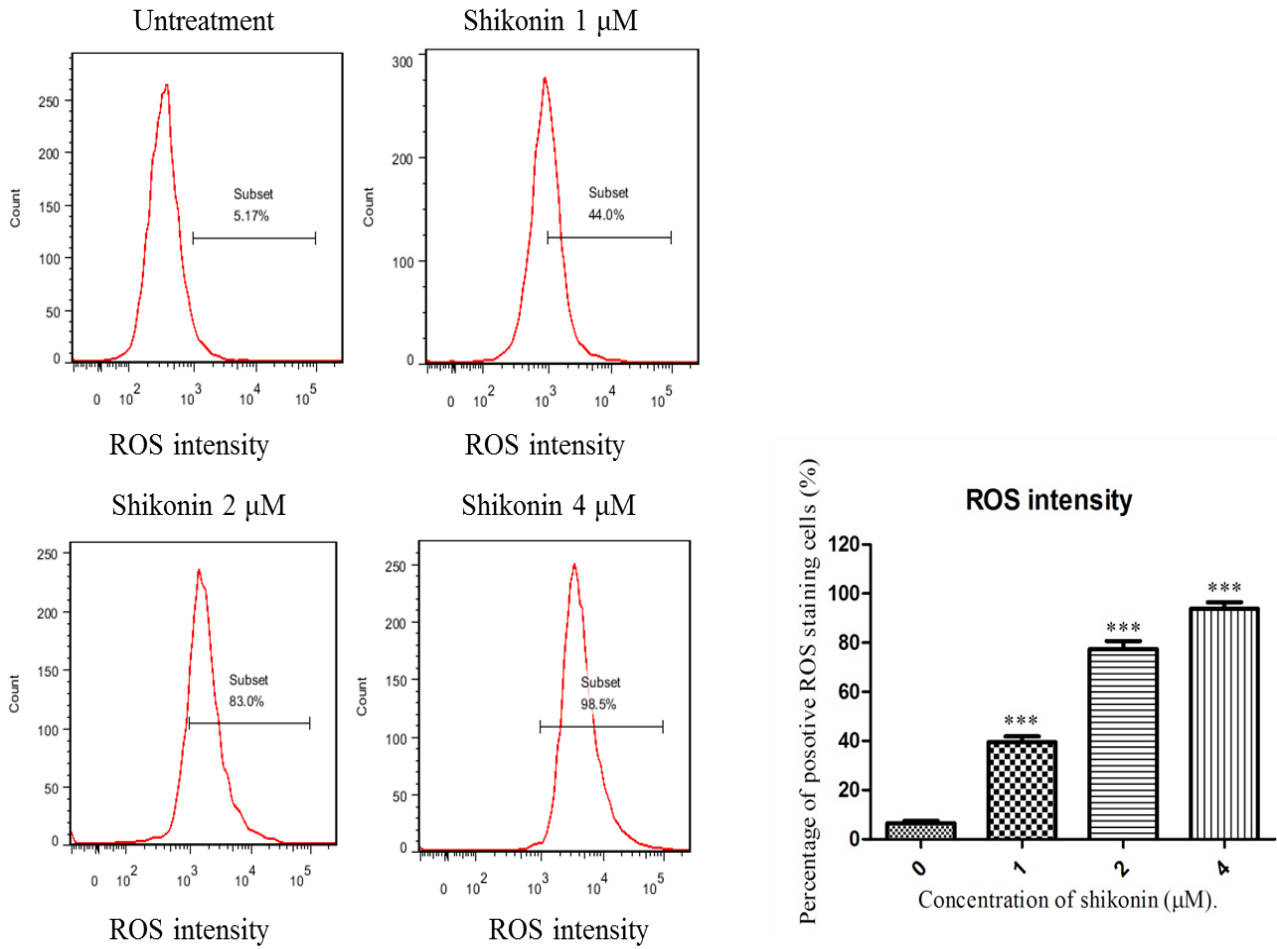
The generation of ROS was increased by the treatment of shikonin. After cultured with different concentration of shikonin for 2 h, the intensity of ROS was greatly detected. Shikonin can greatly elevate the generation of ROS in HLE B-3 cells. * *p* < 0.05, ** *p* < 0.01, *** *p* < 0.001.

To elucidate whether shikonin-induced apoptosis on HLE B-3 cells is due to oxidative stress, cells were exposed to different concentration of shikonin for 2 h and the level of ROS was measured by using CM-H_2_DCFDA, a general oxidative stress indicator. As shown in Figure 6, compared with the shikonin untreated group, the ROS generation in the treated group was sharply increased. These interesting results suggest that ROS generation is an early response of cells to the treatment of shikonin.

**Figure 7:**
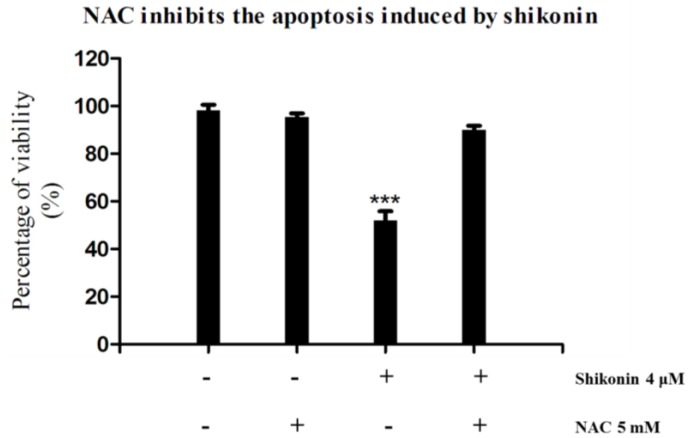
Co-treatment of NAC can significantly offset the anti-proliferation effect of shikonin in HLE B-3 cells. * *p* < 0.05, ** *p* < 0.01, *** *p* < 0.001.

Furthermore, we tried to demonstrate whether ROS plays an essential role in the anti-proliferation effect of shikonin in HLEs. NAC, a specific inhibitor of ROS was applied as a co-treatment with shikonin. From Figure 7, we see that the additions of NAC completely blocked the anti-proliferation effect of shikonin. Taken together, these results suggested that ROS generation is a quick response of cell to the treatment of shikonin, and it is essential for shikonin to inhibit the growth of HLEs.

### 2.5. MAPK Pathway was Involved in Shikonin-Induced Apoptosis

**Figure 8:**
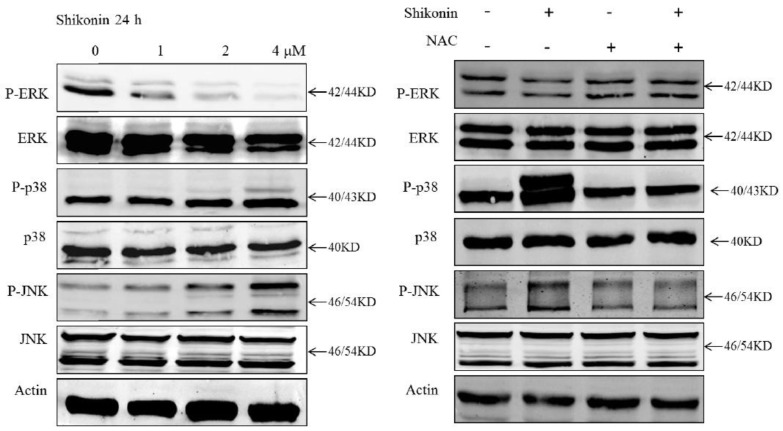
MAPK pathway was involved in shikonin-induced apoptosis. NAC totally blocked the suppression of ERK and activation of JNK and p38.

It has been extensively proved that the mitogen-activated protein kinases (MAPK) pathway plays a critical role in controlling cell proliferation and death. It has been reported that shikonin can regulate JNK and p38 activation in chronic myelocytic leukemia [16]. To determine the effect of shikonin on the MAPK pathway in HLE B-3 cells, we investigated the activation of three kinases of this pathway. As shown in Figure 8, all three kinases of the MAPK pathway were affected. The phosphorylation of extracellular signal-regulated kinase (ERK) was suppressed, while phosphorylation of c-Jun N-terminal kinases (JNK) and p38 were activated. Furthermore, the suppression of ERK and activation of JNK and p38 was totally blocked by NAC. These suggest that the MAPK pathway was associated with shikonin-induced apoptosis which could be blocked by ROS scavengers.

## 3. Experimental Section

### 3.1. Chemicals

Shikonin powder and *N*-acetyl-L-cysteine (NAC) were purchased from Sigma Aldrich (St. Louis, MO, USA). Pan-caspase inhibitor was purchased from BD Pharmingen (San Jose, CA, USA). Antibodies against Bcl-2 and Bax were purchased from Santa Cruz Biotechnology (Dallas, TX, USA). And antibodies of caspase-3/7, phospho-ERK, total ERK, phospho-JNK, total JNK, phospho-p38, total p38, PARP, were purchased from Cell Signaling Technology (Danvers, MA, USA). The fluorescein-conjugated secondary antibodies were purchased from Odyssey (LI-COR, Belfast, ME, USA).

### 3.2. Cell Line and Cell Culture

HLE B-3 was purchased from ATCC and was the only human lens epithelium cell line which ATCC provided. Cells were cultured with RPMI 1640 medium supplemented with 20% fetal bovine serum (Gibco, Big Cabin, Oklahoma, ME, USA), 100 U/mL penicillin and 100 μg/mL streptomycin (Gibco), and cultivated at 37 °C with an incubator supplying 5% CO_2_ [19].

### 3.3. 3-(4,5-Dimethylthiazol-2-yl)-2,5-diphenyltetrazolium Bromide (MTT) Assay

HLE B-3 cells were seeded on a 96 well microplate with 5000 cells/well, and were cultured overnight for cell adhesion. Shikonin was added and was incubated for 24, 48 or 72 h with vehicle Dimethyl sulfoxide (DMSO) as the control. Each dosage was repeated as triplicate. Ten μL of MTT (5 mg/mL) was added to every well and the plate was placed in the incubator for 4 h. Then 100 μL of formazan crystals solubilizing solution (10% SDS and 0.01%M HCL) was added to each well and the plate was further incubated at 37 °C for another 4 h to dissolve the formazan crystals. Finally, after shaking 10 min at room temperature, the absorbance of each well was measured at 570 nm using a microplate reader (Tecan, Morrisville, NC, USA). The cell viability is calculated by dividing the absorbance of treated cells by that of vehicle control.

### 3.4. Assessment of Apoptosis Levels by Annexin V/PI Staining

According to the manufacturer’s (BD Pharmingen) protocol, cells were harvested, washed, and double-stained with Annexin V/PI for 15 min at room temperature in dark. Then apoptotic cells were quantitatively counted by a flowcytometry. Early stage of apoptotic cells were stained as Annexin V-positive and PI-negative, while the late stage apoptotic cells were stained as Annexin V-positive and PI-positive.

### 3.5. Analysis of Mitochondrial Membrane Potential (MMP)

JC-1 staining (BD Pharmingen) was applied to determine the MMP level of cells. After treatment, cells were harvested, washed, and stained with JC-1 for 15 min in the incubator at 37 °C. Then cells were washed again and re-suspended with regular medium. The change in MMP of the cells was measured by flow cytometer followed with the manufacturer’s protocol. In brief, the fluorescence emission spectrum of JC-1 is dependent on the concentration of the JC-1 forms. There are two forms of JC-1 which include the monomer and the aggregate forms. The monomer form of JC-1 represents the loss of MMP and yields a green fluorescence signal, while the increase of red fluorescence indicates that JC-1 aggregates together in normal hyperpolarized mitochondria.

### 3.6. Western Blot Analysis

After treatment, ice-cold radioimmunoprecipitation assay (RIPA) lysis buffer with protease and phosphatase inhibitors was add to the cells for total cellular protein extraction. The concentration of the total cell lysate was determined by Bio-Rad detergent-compatible (DC™) colorimetric protein assay kit and proteins were denatured by heating 95 °C for 5 min. The prepared samples were loaded onto a 10% SDS-PAGE gel to separate. After electrophoresis, the proteins on SDS-PAGE gel were transferred to a Nitrocellulose (NC) membrane at 300 mA for 2 h. Membranes were blocked with 5% milk without fat in TBST for 1 h at room temperature. Primary antibodies (1:1000 dilutions for Cell Signaling antibodies; 1:500 dilutions for Santa Cruz antibodies) were incubated overnight at 4 °C. After washing by Tris Buffered Saline with Tween^®^ 20 (TBST) three times (5 min/time), secondary fluorescent antibody (1:10000 dilutions) was added to membrane at room temperature for 1 h. Actin was used as loading control and for normalization. The signal intensity of the membranes was detected by Odessy (LI-COR, Belfast, ME, USA).

### 3.7. Detection of ROS Generation

ROS was analyzed by using dichlorofluorescein diacetate (CM-H_2_DCFDA, Invitrogen, Carlsbad, CA, USA) fluorescent probe staining, which is a specific superoxide tracing dye. Cells were pretreated with CM-H_2_DCFDA 20 μM 30 min at 37 °C prior to treatment. And then cells were incubated for the indicated times with shikonin and vehicle control, harvested and re-suspended in phosphate buffered saline (PBS). Fluorescence was measured with flowcytometry with excitation and emission settings of 488 and 525 nm, respectively.

### 3.8. Statistical Analysis

All of the data are expressed as mean ± the standard error of the mean (SEM) of three individual experiments. Differences between groups were determined by one way analysis of variance (ANOVA), followed by Dunnet’s test to compare the treated groups with the control, with *p* values < 0.05 was considered significant.

## 4. Discussion and Conclusions

After cataract surgery, the residual HLEs at the equator and under the anterior lens capsule proliferate and migrate onto the posterior capsule and undergo epithelial-mesenchymal transition, resulting in the formation of fibroblasts, myofibroblasts, and extracellular cell matrix, and finally, PCO [20].

PCO is the most frequent complication and the primary reason of visual decrease after surgery [21]. Although numerous studies have been taken to prevent or overcome the problem of PCO [22], all of these methods have deficiencies, even the usual treatment: YAG laser, which not only requires expensive laser equipment but can cause complications such as cystoid macular edema, retinal detachment, and glaucoma [23]. Therefore, finding some new methods which is not only effective but has lower side effect is required. Low toxic natural products from herbs may provide more alternative way for preventing or treating PCO.

Shikonin is a major component of *Lithospermum erythrorhizon*, a herbal medicine with various biological activities [24]. It has been reported that shikonin possessed powerful inhibitory effects on the proliferation of various cancer cells. It is also quite safe, which has been demonstrated *in vivo* [14]. In this study, we found that the inhibition of shikonin on the growth of HLE B-3 occurs in a dose and time dependent manner, and shikonin inhibit the proliferation of HLEs through the induction of apoptosis which could be a novel therapy approach for prevention of PCO [8].

In this apoptotic process, ROS generation was remarkably up-regulated and played a necessary role. Once the cells fail to remove the excessive ROS, this will lead to mitochondrial disruption and cell death, which was proved by an MMP assay. During mitochondrial dysfunction, Bcl-2 family members cause outer membrane permeabilization, which subsequently allows cytochrome C that is sequestered in the mitochondrial intermembrane space to be released into the cytosol and activate caspases [25]. We found that by treating with shikonin, the ratio of anti-apoptotic protein Bcl-2 and pro-apoptotic protein Bax was greatly decreased, and both caspase-3 and 7 were activated. The co-treatment of pan-caspase inhibitor largely blocked the apoptosis. Therefore, shikonin-induced apoptosis is through a caspases dependent pathway.

The MAPK superfamily consists of three main protein kinase families: ERKs, JNK and the p38. It has been reported that they are all closely correlated with the growth of cells and plays an essential role in regulating the intracellular metabolism and responding to external stress [26]. It has been reported before that shikonin exerted anti-inflammatory action by suppression the ERK *in vivo* [27]. Moreover, it has been shown that ERKs are important for cell survival, whereas JNKs and p38 were associated with stress responsive and thus involved in apoptosis [28], which corresponds with our findings. Therefore, all three kinases of the MAPK path play important roles in shikonin-induced apoptosis.

In sum, we have demonstrated that shikonin-induced apoptosis occurs through ROS generation and a caspase activation dependent pathway. The MAPK pathway was also involved. This may provide a new avenue for treating PCO patients in the future. However, the present *in vitro* study is only preliminary. Further detailed and intensive researches *in vivo* and even the clinic should be undertaken to elucidate the benefits and effects of shikonin on PCO.
